# Characteristics and hematological indicators predictors of immunotherapy response in advanced non-small cell lung cancer

**DOI:** 10.1186/s12885-026-16302-w

**Published:** 2026-06-11

**Authors:** Nan Zhao, Xinyu Wu, Wensi Zhao, Xiang Cheng, Hua He, Dedong Cao

**Affiliations:** 1 Department of Oncology, Renmin Hospital of Wuhan University, 238 Jiefang Road, Wuchang District, Wuhan, 430000 China; 2 Taikang Tongji Hospital of Wuhan, Wuhan, China; 3 Big Data Health and Medical Research and Application Center of Wuhan University, Wuhan, China; 4 Department of Medical Records Management, Renmin Hospital of Wuhan University, Wuhan, China

**Keywords:** NSCLC, Immunotherapy, Biomarkers, Inflammatory markers, Predictive factors

## Abstract

**Objective:**

Immune checkpoint inhibitors (ICIs) have significantly improved the treatment outcomes for advanced non-small cell lung cancer (NSCLC), but patient benefits vary individually. Therefore, identifying biomarkers to predict the efficacy and prognosis of immunotherapy is crucial. Hematological markers such as the neutrophil-to-lymphocyte ratio (NLR), platelet-to-lymphocyte ratio (PLR), albumin-bilirubin (ALBI) score, and lactate dehydrogenase (LDH) levels may correlate with tumor prognosis. This study aimed to evaluate the prognostic value of these hematological and clinical markers in advanced NSCLC patients treated with ICIs, providing a basis for individualized treatment strategies.

**Methods:**

This retrospective study included NSCLC patients treated with ICIs at the Tumor Centers of Renmin Hospital of Wuhan University and Macheng City People’s Hospital between January 2021 and December 2023. Clinical data such as gender, age, ECOG PS score, clinical stage, and treatment details were collected. Patients were stratified based on NLR, PLR, LDH, and ALBI scores. ROC curve analysis assessed the predictive capacity of these markers for mortality risk. Chi-square tests, logistic regression, Kaplan-Meier survival analysis, and log-rank tests were used to analyze short-term efficacy, immune-related adverse events (irAEs), progression-free survival (PFS), and overall survival (OS). Cox regression identified independent prognostic factors for PFS and OS.

**Results:**

A total of 198 advanced NSCLC patients (median follow-up: 28.1 months) were included. Median PFS (mPFS) and OS (mOS) were 7.7 months (95% CI: 6.9 ~ 8.5) and 20.1 months (95% CI: 18.2 ~ 21.9), respectively. ROC analysis demonstrated significant predictive value for baseline NLR, PLR, ALBI, and LDH in mortality risk (AUC: 0.804, 0.694, 0.684, 0.726, respectively). Efficacy analysis revealed PD-L1 positivity (OR = 0.361, 95% CI: 0.161 ~ 0.808, *P* = 0.013) and ALBI < -2.68 (OR = 2.524, 95% CI: 1.148–5.552, *P* = 0.021) as independent predictors of objective response rate (ORR: 25.8%), while camrelizumab significantly reduced response rates (OR = 0.157, *P* = 0.006).

Baseline NLR, PLR, ALBI, and LDH significantly stratified PFS (*P* < 0.05): low NLR (8.7 vs. 7.1 months, *P* = 0.001), low PLR (7.9 vs. 7.4 months, *P* = 0.007), low ALBI (8.1 vs. 7.0 months, *P* = 0.028), and low LDH (9.5 vs. 7.1 months; 46.3% risk reduction, *P* < 0.001). In the adenocarcinoma subgroup, LDH remained an independent prognostic factor (9.5 vs. 6.8 months, *P* = 0.032). For squamous cell carcinoma, low NLR (9.1 vs. 7.1 months, *P* = 0.002), low PLR (10.2 vs. 7.4 months, *P* = 0.002), low ALBI (8.7 vs. 7.1 months, *P* = 0.022), and low LDH (9.1 vs. 7.5 months, *P* = 0.002) were significant. Baseline markers also predicted OS: low NLR (21.4 vs. 17.5 months, *P* < 0.001), low PLR (20.4 vs. 17.7 months, *P* = 0.009), and low LDH (21.4 months; 42.5% risk reduction, *P* < 0.001). Subtype analysis showed adenocarcinoma benefited from low NLR (21.4 vs. 16.5 months, *P* = 0.013) and low LDH (20.1 vs. 17.5 months, *P* = 0.021), while squamous carcinoma relied on low NLR (21.4 vs. 16.8 months, *P* = 0.008), low PLR (21.8 vs. 20.1 months, *P* = 0.024), low ALBI (21.4 vs. 18.2 months, *P* = 0.047), and low LDH (24.6 vs. 19.8 months, *P* = 0.001).

Multivariate Cox analysis identified radiotherapy, NLR, and LDH as independent predictors of PFS: Radiotherapy reduced risk by 37.5% (HR = 0.625, *P* = 0.004); NLR ≥ 3.72 (HR = 1.642) and LDH ≥ 226.5 U/L (HR = 1.821) increased risk by 64.2% and 82.1% (*P* < 0.05). For OS, adenocarcinoma (HR = 0.361) and squamous carcinoma (HR = 0.350) reduced mortality risk by 63.9% and 65.0% (*P* < 0.05), while NLR ≥ 3.72 (HR = 1.536), and LDH ≥ 226.5 U/L (HR = 1.728) increased risk by 53.6% and 72.8% (*P* < 0.05). LDH ≥ 226.5 U/L (HR = 2.372) stained highest risk in squamous carcinoma (*P* < 0.05).

**Conclusions:**

Baseline hematological markers (NLR, PLR, ALBI, LDH) are valuable predictors of efficacy and prognosis in advanced NSCLC patients undergoing immunotherapy. Their prognostic roles vary by pathological subtype: squamous carcinoma relies more on inflammatory markers, while adenocarcinoma emphasizes metabolic markers and genetic mutations. This study provides a hematological biomarker-based stratification framework for individualized immunotherapy decisions in advanced NSCLC, offering critical guidance for optimizing treatment and prognosis management.

**Supplementary Information:**

The online version contains supplementary material available at 10.1186/s12885-026-16302-w.

## Introduction

Lung cancer remains a major threat to global public health, with its epidemiological features showing a persistent worsening trend. According to data from the International Agency for Research on Cancer (IARC), there are over 2.2 million new lung cancer cases annually worldwide, accounting for 12.4% of malignant tumors; and 1.8 million deaths, representing 18% of cancer-related deaths [1]. Pathologically, non-small cell lung cancer (NSCLC) predominates (85%), with adenocarcinoma subtypes continuing to rise [2]. With advances in modern medical technology, early-stage lung cancer patients can achieve favorable prognosis through surgical resection combined with adjuvant therapy, with 5-year survival rates reaching 60% to 80%. However, clinical data indicate that approximately 70% of NSCLC patients are diagnosed at stage IIIB or IV [3]. Traditional chemotherapy regimens (e.g., platinum combined with pemetrexed) can partially alleviate symptoms, but the median overall survival (mOS) remains only 8–12 months, accompanied by significant toxicities such as bone marrow suppression and gastrointestinal reactions [4]. Targeted therapies (e.g., EGFR-TKIs, ALK inhibitors) have achieved breakthroughs in driver gene-positive patients, but their coverage is limited (only about 30% of NSCLC patients have actionable targets) and drug resistance issues restrict widespread application [5]. This dilemma has prompted researchers to turn their attention to immunotherapy.

The advent of immune checkpoint inhibitors (ICIs) has transformed the landscape of traditional cancer treatment, introducing novel therapeutic approaches to clinical practice. Their mechanism of action focuses on relieving immunosuppressive signals in the tumor microenvironment (TME): PD-1/PD-L1 pathway inhibitors block the binding of PD-1 receptors on T cells to PD-L1 ligands on tumor cells, restoring effector T cell anti-tumor activity; while CTLA-4 inhibitors enhance activation signals during the T cell priming phase, expanding the immune response spectrum [6]. The KEYNOTE-024 study showed that in advanced NSCLC patients with high PD-L1 expression (tumor proportion score TPS ≥ 50%), pembrolizumab monotherapy significantly prolonged mOS compared to chemotherapy (30.0 vs. 14.2 months, HR = 0.63), with a nearly 50% reduction in grade 3 or higher adverse events [7]. The IMpower150 trial demonstrated that in non-squamous NSCLC, the triple regimen of atezolizumab, bevacizumab, and chemotherapy significantly improved outcomes, with an objective response rate of 64%, benefiting even patients with brain metastases [8]. Based on these breakthroughs, ICIs have been elevated from second-line to first-line standard therapy for advanced NSCLC.

Nevertheless, the widespread application of immunotherapy still faces numerous challenges. Clinical data show that only about 50% of patients respond to ICIs, with long-term tumor remission achieved in only 10%-15%. Meanwhile, approximately 10%-15% of patients may experience severe immune-related adverse events (irAEs), which can lead to long-term sequelae or even life-threatening conditions. Therefore, selecting patients who can benefit from ICIs and preventing/managing potential irAEs have become key research priorities and challenges.

In current clinical practice, predictive markers are mainly divided into two categories: tumor-intrinsic markers and host-related markers. PD-L1 TPS, as the most widely used marker, has been extensively applied in treatment decision-making for advanced NSCLC patients [9, 10]. Generally, patients with higher PD-L1 TPS levels have higher response rates. However, the predictive ability of PD-L1 TPS is not absolute; some patients with high PD-L1 TPS do not benefit, while some with low or negative expression respond [11, 12]. Additionally, tumor mutational burden (TMB) has garnered significant research interest as a potential biomarker [13]. Multiple studies indicate that high TMB may correlate with higher response rates [14, 15]. Beyond biological markers, patient clinical features may also play important roles in efficacy prediction, such as gender [16], performance status (ECOG PS) [17, 18], body mass index (BMI) [19, 20], specific sites of tumor metastasis [16], and concomitant medications (e.g., antibiotics and corticosteroids [21–23]). In recent years, the pathophysiology of irAEs and their correlation with individual patient characteristics have become research hotspots. Some studies suggest that irAEs occurrence may correlate with higher response rates, but the specific mechanisms remain unclear, and balancing reduced irAEs risk while ensuring efficacy is a pressing clinical issue.

In recent years, systemic inflammation indicators based on peripheral blood have emerged as research hotspots for prognosis assessment due to their real-time, dynamic, and non-invasive advantages. Particularly in NSCLC patients, inflammatory markers in the blood are thought to be closely related to the formation of immunosuppressive microenvironments [12]. Studies show that elevated neutrophil-to-lymphocyte ratio (NLR) and platelet-to-lymphocyte ratio (PLR) often predict shorter progression-free survival (PFS) and overall survival (OS) [24–26]. A study involving 187 patients found that NLR < 5 was associated with improved PFS and OS, and PLR < 200 with PFS, OS, ORR, and DCR [27], suggesting NLR and PLR as potential predictive markers for ICIs efficacy. Lactate dehydrogenase (LDH) and albumin-bilirubin index (ALBI) scores are also considered important prognostic factors. LDH, as a key marker of tumor cell metabolism, has been shown to correlate with cancer malignancy and patient prognosis [28]. Elevated LDH (> 250 U/L) indicates hypoxic microenvironment and active tumor metabolism, associated with primary resistance to ICIs (ORR reduced by 62%) [29]. Kinoshita’s team found that preoperative ALBI grade was significantly associated with postoperative recurrence in NSCLC, with ALBI grades 2/3 as potential independent prognostic factors for disease-free survival (DFS) and OS [30], suggesting it as a novel predictive tool for immunotherapy.

Several studies have demonstrated that squamous cell carcinoma and adenocarcinoma exhibit distinct tumor microenvironments: squamous cell carcinoma is typically associated with smoking, higher tumor mutational burden, and a more inflamed immune profile, while adenocarcinoma more frequently harbors driver mutations (EGFR, ALK, KRAS) that may modulate ICI response. Given these biological differences, we hypothesized that the predictive value of hematological markers might differ between subtypes.

Given the above research background, this study employs retrospective analysis to systematically evaluate clinical data from advanced NSCLC patients receiving ICIs, focusing on the clinical value of NLR, PLR, LDH, and ALBI in prognosis assessment. Subgroup analyses by pathological type (adenocarcinoma and squamous carcinoma) were conducted to provide evidence-based support for selecting immunotherapy beneficiaries and optimizing treatment decisions, while offering new insights for early irAEs warning.

## Materials and methods

### General information

This retrospective study collected data from 198 NSCLC patients treated with ICIs at the Tumor Centers of Renmin Hospital of Wuhan University and Macheng People’s Hospital between January 2021 and December 2023. The study was approved by the Clinical Research Ethics Committee of Renmin Hospital of Wuhan University (Ethics Approval No.: WDRY2020F048).

#### Inclusion criteria

1. Pathologically or histologically confirmed non-small cell lung cancer (NSCLC);
2. Advanced lung malignancy (stage IIIB/IV or recurrent disease not amenable to curative surgery or radical radiotherapy intended for early-stage disease); palliative radiotherapy for metastatic lesions was allowed;
3. ECOG PS score: 0–2;
4. Received at least 2 cycles of immune checkpoint inhibitor therapy;
5. Complete baseline data: enhanced CT/PET-CT within 30 days before treatment, pathological subtype and staging evidence, blood test indicators, etc.

#### Exclusion criteria

1. History of other malignancies within 5 years (excluding non-melanoma skin cancer/cervical carcinoma in situ);
2. Presence of any of the following: active autoimmune disease (requiring immunosuppressants, e.g., systemic lupus erythematosus, myasthenia gravis); uncontrolled systemic infection (CRP > 3 times normal upper limit with fever); interstitial lung disease (CT-confirmed pulmonary interstitial fibrosis > 20%);
3. Patients with fewer than two treatment cycles;
4. Patients lacking complete clinical data.

### Data collection

Data collection encompassed outpatient and inpatient clinical information, including: (1) Demographic and baseline characteristics: age, gender, smoking history, comorbidities, etc.; (2) Tumor characteristics: histological subtype (adenocarcinoma/squamous carcinoma/other), molecular typing (PD-L1 TPS detection platform and antibody, TMB value, driver gene mutations), tumor TNM staging, primary lesion maximum diameter (pre-treatment CT measurement), metastatic burden, etc.; (3) Treatment parameters: immunotherapy regimen (drug name and dose, line of therapy, combination regimen, cycles); (4) Laboratory indicators: complete blood count (absolute neutrophil count (ANC), absolute lymphocyte count (ALC), platelet count (PLT), absolute monocyte count (AMC), etc.), liver function (alanine aminotransferase (ALT), aspartate aminotransferase (AST), albumin (ALB), total bilirubin (TBIL)), inflammatory markers (C-reactive protein, lactate dehydrogenase (LDH), ferritin), renal function (creatinine, estimated glomerular filtration rate (eGFR)); (5) Imaging and efficacy assessment (6) Safety data: immune-related adverse reactions, treatment interruption/termination records; (7) Survival follow-up: Primary endpoints: PFS: from treatment initiation to radiological progression/death; OS: from treatment initiation to all-cause death.

Biomarker definitions: (1) NLR (neutrophil-to-lymphocyte ratio) = absolute neutrophil count (×10⁹/L) divided by absolute lymphocyte count (×10⁹/L); (2) PLR (platelet-to-lymphocyte ratio) = platelet count (×10⁹/L) divided by absolute lymphocyte count (×10⁹/L); (3) ALBI (albumin-bilirubin) score = [log₁₀ bilirubin (µmol/L) × 0.66] + [albumin (g/L) × − 0.085].

### Treatment regimens

This study selected patients receiving systemic ICIs therapy as subjects, dividing them into four subgroups based on regimens: ICIs monotherapy, ICIs combined with chemotherapy, ICIs combined with targeted therapy, and ICIs combined with chemotherapy and targeted therapy. Regimen selection was based on individualized clinical features and the attending physician’s professional judgment. All immune checkpoint inhibitors were administered per label/approved indications under the supervision of the attending physician. Dosing reductions or delays were at the physician’s discretion based on patient tolerance.

Radiotherapy in the cohort was exclusively palliative, administered for symptomatic metastases (bone, brain, or painful soft tissue lesions) or for oligoprogression. No patient received radical-intent radiotherapy (e.g., stereotactic body radiation therapy with curative dose > 60 Gy for early-stage disease).

### Efficacy evaluation methods

According to the Response Evaluation Criteria in Solid Tumors (RECIST 1.1), each patient underwent chest enhanced CT every 8–12 weeks for efficacy assessment. Data follow-up cutoff was December 1, 2024.

This study employed the Response Evaluation Criteria in Solid Tumors (RECIST 1.1) for systematic evaluation. All patients underwent baseline chest enhanced CT (slice thickness ≤ 2 mm, contrast agent iohexol 100 mL) to identify target lesions (up to 5, ≤ 2 per organ), with imaging re-evaluated every 8–12 weeks during treatment. Follow-up endpoint was set as December 1, 2024. Cases lost to follow-up at the last visit were treated as censored data in statistical analysis. Efficacy was assessed according to international standards, categorized into four levels: (1) Complete Response (CR): Imaging shows complete disappearance of all target lesions, complete regression of non-target lesions, normalization of tumor markers, and no new lesions; (2) Partial Response (PR): Sum of target lesion diameters reduced by ≥ 30% from baseline, with no progression in non-target lesions; (3) Stable Disease (SD): Changes in lesion diameters do not meet PR criteria but also do not meet progression criteria; (4) Progressive Disease (PD): Sum of target lesion diameters increased by ≥ 20%, or clear progression in non-target lesions, or new lesions detected. All efficacy determinations were objectively based on imaging and laboratory results.

### Statistical methods

This study used SPSS 27.0 and R 4.3.1 for data analysis, with GraphPad Prism 10 for visualization. Normally distributed continuous variables are presented as mean ± standard deviation ($\:\stackrel{-}{\mathrm{x}}$±s), and categorical variables as frequencies (percentages). Inter-group differences were compared using chi-square tests or Fisher’s exact test (when expected frequency < 5). ROC curve analysis determined optimal cut-off values for peripheral blood markers and calculated corresponding area under the curve (AUC) and 95% confidence intervals. For survival analysis, Kaplan-Meier method was used to plot PFS and OS curves, with log-rank tests for inter-group comparisons. Cox proportional hazards regression model was employed for multivariate prognostic factor assessment. All statistical analyses in this study used two-sided tests, with *P* < 0.05 considered statistically significant.

### irAE assessment and attribution

Adverse events were graded according to CTCAE version 5.0. irAEs were defined based on the following criteria: (1) typical clinical presentation of immune-related toxicity; (2) temporal association with ICI administration; (3) response to corticosteroids or immunosuppressive therapy.

## Results

### Patient baseline characteristics

**Fig. 1:**
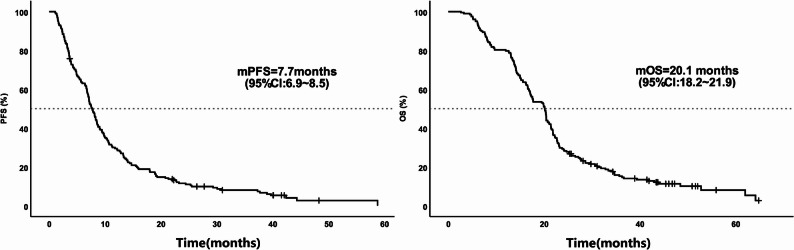
PFS and OS Survival Curves for 198 NSCLC Patients

This study included a total of 198 patients with advanced NSCLC from the Oncology Centers of Renmin Hospital of Wuhan University and Macheng People’s Hospital (Table 1). Among them, male patients accounted for 78.28%, and female patients for 21.72%. Patients under 65 years old comprised 53.03%, and those with ECOG PS scores of 0–1 accounted for 85.35%. In terms of clinical staging, stage IV patients accounted for 70.20%, with adenocarcinoma being the predominant pathological type (55.05%), followed by squamous cell carcinoma (41.92%). Regarding treatment, most patients received immunotherapy combined with chemotherapy (69.19%), with tislelizumab, camrelizumab, and sintilimab being commonly used immunotherapy drugs (accounting for 30.30%, 25.76%, and 24.24%, respectively), and 55.56% of patients received radiotherapy. PD-L1-positive patients for 22.22%, And gene mutation-positive patients accounted for only 18.18%. Details of gene mutations are shown in Supplementary Table S1. In terms of hematological immune response markers, patients with NLR < 3.72 accounted for 51.01%, PLR < 207.43 for 52.02%, ALBI score <-2.68 for 59.60%, and LDH < 226.50 for 41.41%. As of December 1, 2024, 176 patients had died during follow-up, with a median follow-up time of 28.1 months, median PFS of 7.7 months (95% CI = 6.9 ~ 8.5 months), and median OS of 20.1 months (95% CI = 18.2 ~ 21.9 months) (Fig. 1).

**Table 1:** Patient Baseline Characteristics

	Characteristic	Cases (%)
Sex	Male	155(78.28)
	Female	43(21.72)
Age	≥ 65	93(46.97)
	<65	105(53.03)
Pathological Type	Adenocarcinoma	109(55.05)
	Squamous Carcinoma	83(41.92)
	Other*	6(3.03)
PS Score	0 ~ 1	169(85.35)
	2	29(14.65)
Clinical Stage	Ⅲ	59(29.80)
	Ⅳ	139(70.20)
Efficacy Evaluation	PR	51(25.76)
	SD	111(56.06)
	PD	36(18.18)
PD-L1 Expression†	Negative	154(77.78)
	Positive	44(22.22)
Gene Mutation	Absent	162(81.82)
	Present	36(18.18)
Line of Therapy	1	78(39.39)
	2	73(36.87)
	3	27(13.64)
	≥ 4	20(10.10)
Immunotherapy Drug	Camrelizumab	51(25.76)
	Pembrolizumab	18(9.09)
	Tislelizumab	60(30.30)
	Sintilimab	48(24.24)
	Other	21(10.61)
Treatment Regimen	Immunotherapy	34(17.17)
	Immuno + Chemo	137(69.19)
	Immuno + Targeted	6(3.03)
	Immuno + Chemo + Targeted	21(10.61)
Radiotherapy	Present	110(55.56)
	Absent	88(44.44)
Brain Metastasis	Absent	151(76.26)
	Present	47(23.74)
Bone Metastasis	Absent	133(67.17)
	Present	65(32.83)
NLR	<3.72	101(51.01)
	≥ 3.72	97(48.99)
PLR	<207.43	103(52.02)
	≥ 207.43	95(47.98)
ALBI	<-2.68	118(59.60)
	≥-2.68	80(40.40)
LDH	<226.50	82(41.41)
	≥ 226.50	116(58.59)

*Other refers to large cell carcinoma

†PD-L1 positive defined as TPS ≥ 1%

### ROC curve analysis

**Fig. 2:**
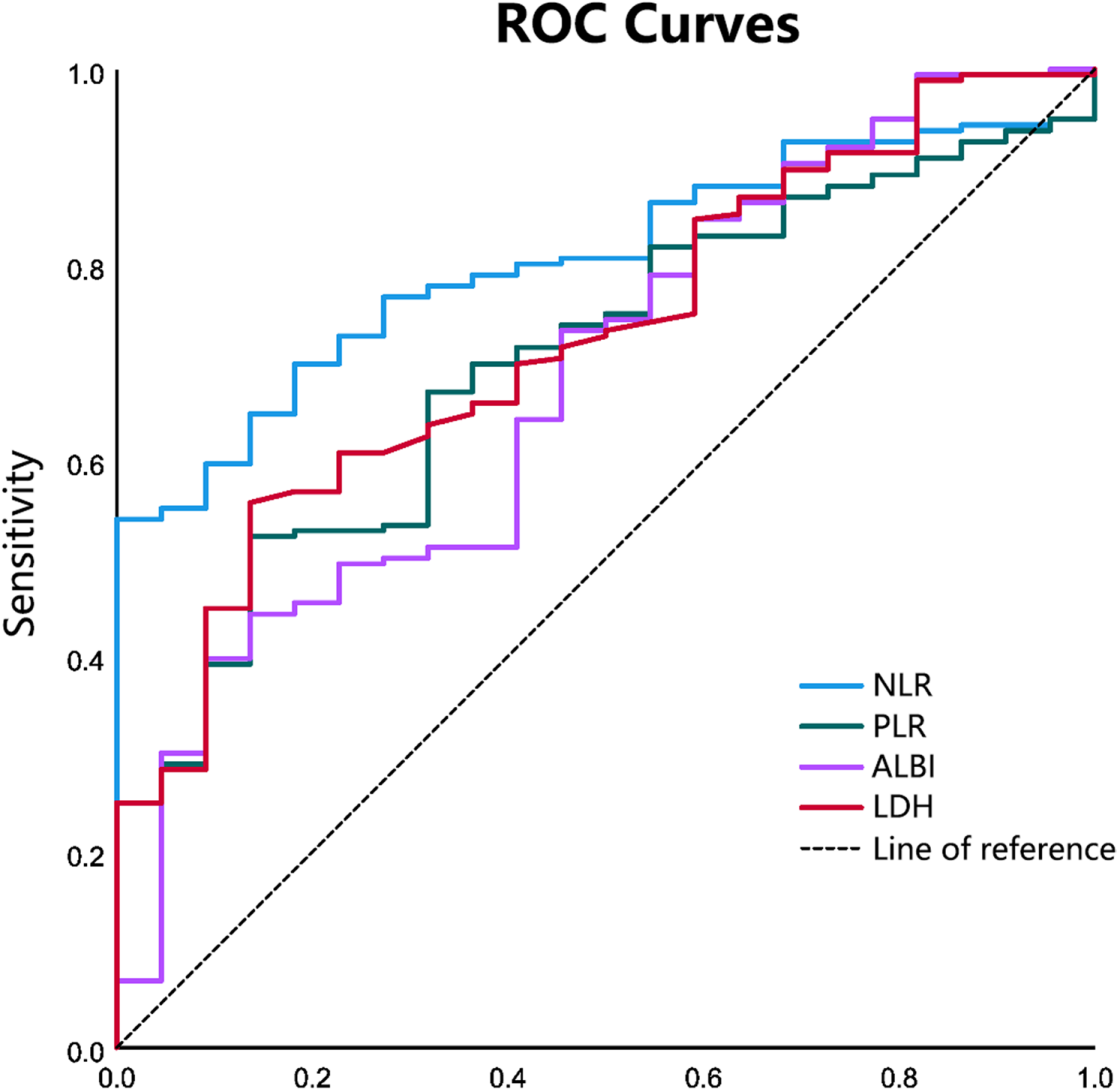
ROC Curves of Baseline NLR, PLR, ALBI, and LDH for Predicting Overall Survival in Advanced Lung Cancer Patients Treated with ICIs

This study performed ROC curve analysis on the overall survival of patients (Fig. 2) to evaluate the discriminative ability of NLR, PLR, ALBI, and LDH in predicting mortality risk. The analysis results showed that the areas under the curve (AUC) for NLR, PLR, ALBI, and LDH were 0.804, 0.694, 0.684, and 0.726, respectively.

The optimal cut-off values calculated using the Youden index were: NLR 3.72, PLR 207.43, ALBI − 2.68, LDH 226.5 U/L. Based on these cut-off values, patients were divided into low-level groups (L-group) and high-level groups (H-group), with further exploration of their correlations with clinical efficacy and survival prognosis.

### Analysis of factors influencing short-term efficacy

By the end of follow-up, the best treatment response assessments for the 198 included patients showed: PR in 51 cases (25.8%), SD in 111 cases (56.1%), PD in 36 cases (18.2%). Accordingly, the objective response rate (ORR) for the entire cohort was 25.8%, and the disease control rate (DCR) was 81.9%. For efficacy analysis, patients were divided into two subgroups: tumor response group (PR patients, *n* = 51) and non-response group (SD + PD patients, *n* = 147).

#### Analysis of short-term efficacy factors in the full cohort

This study included 198 patients, of which 51 (25.76%) achieved PR (Table 2). Univariate analysis showed that PD-L1 positive expression (40.91% vs. 21.43%, χ²=6.791, *P* = 0.009), absence of bone metastasis (30.08% vs. 16.92%, χ²=3.949, *P* = 0.047), PLR < 207.43 (32.04% vs. 18.95%, χ²=4.429, *P* = 0.035), and ALBI <-2.68 (31.36% vs. 17.50%, χ²=4.787, *P* = 0.029) were significantly associated with higher PR rates. There were also significant differences in PR rates among different immunotherapy drugs (χ²=12.862, *P* = 0.012), with pembrolizumab having the highest PR rate (50.00%) and camrelizumab the lowest (13.73%).

**Table 2:** Correlation Analysis Between Clinical Characteristics and Short-Term Efficacy in the Full Cohort

Characteristic		Total	Tumor Response Group *n*(%)*	Tumor Non-Response Group *n*(%)*	x2	*P* Value	OR (95%CI)	*P* Value
Sex	Male	155	42(27.10)	113(72.90)	0.669	0.413		
	Female	43	9(20.93)	34(79.07)				
Age	<65	105	28(26.67)	77(73.33)	0.097	0.756		
	≥ 65	93	23(24.73)	70(75.27)				
Pathological Type	Adenocarcinoma	109	24(22.02)	85(77.98)	2.402	0.301		
	Squamous Carcinoma	83	26(31.33)	57(68.67)				
	Other	6	1(16.67)	5(83.33)				
PS Score	0 ~ 1	169	47(27.81)	122(72.19)	2.757	0.252		
	2	29	4(13.79)	25(86.21)				
Clinical Stage	Ⅲ	59	20(33.90)	39(66.10)	2.913	0.088	0.954(0.425 ~ 2.139)	0.909
	Ⅳ	139	31(22.30)	108(77.70)				
PD-L1 Expression	Negative	154	33(21.43)	121(78.57)	6.791	0.009	0.361(0.161 ~ 0.808)	0.013
	Positive	44	18(40.91)	26(59.09)				
Gene Mutation	Absent	162	38(23.46)	124(76.54)	2.466	0.116		
	Present	36	13(36.11)	23(63.89)				
Line of Therapy	1	78	18(23.08)	60(76.92)	4.621	0.099		
	2	73	25(34.25)	48(65.75)			0.558(0.248 ~ 1.256)	0.159
	3	27	4(14.81)	23(85.19)			1.549(0.396 ~ 6.068)	0.529
	≥ 4	20	4(20.00)	16(80.00)			0.802(0.193 ~ 3.332)	0.762
Immunotherapy Drug	Other	21	8(38.10)	13(61.90)	12.862	0.012		
	Camrelizumab	51	7(13.73)	44(86.27)			0.157(0.042 ~ 0.59)	0.006
	Pembrolizumab	18	9(50.00)	9(50.00)			0.311(0.109 ~ 0.885)	0.029
	Tislelizumab	60	18(30.00)	42(70.00)			0.681(0.212 ~ 2.181)	0.517
	Sintilimab	48	9(18.75)	39(81.25)			0.258(0.07 ~ 0.952)	0.042
Treatment Regimen	Immunotherapy	34	9(26.47)	25(73.53)	2.197	0.533		
	Immuno + Chemo	137	36(26.28)	101(73.72)				
	Immuno + Targeted	6	0(0.00)	6(100.00)				
	Immuno + Chemo + Targeted	21	6(28.57)	15(71.43)				
Radiotherapy	Present	110	33(30.00)	77(70.00)	2.329	0.127		
	Absent	88	18(20.45)	70(79.55)				
Brain Metastasis	Absent	151	41(27.15)	110(72.85)	0.637	0.421		
	Present	47	10(21.28)	37(78.72)				
Bone Metastasis	Absent	133	40(30.08)	93(69.92)	3.949	0.047	1.955(0.791 ~ 4.831)	0.146
	Present	65	11(16.92)	54(83.08)				
NLR	<3.72	101	30(29.70)	71(70.30)	1.678	0.195		
	≥ 3.72	97	21(21.65)	76(78.35)				
PLR	<207.43	103	33(32.04)	70(67.96)	4.429	0.035	1.620(0.767 ~ 3.418)	0.206
	≥ 207.43	95	18(18.95)	77(81.05)				
ALBI	<-2.68	118	37(31.36)	81(68.64)	4.787	0.029	2.524(1.148 ~ 5.552)	0.021
	≥-2.68	80	14(17.50)	66(82.50)				
LDH	<226.50	82	24(29.27)	58(70.73)	0.902	0.342		
	≥ 226.50	116	27(23.28)	89(76.72)				

*Non-response (SD/PD) = 1, Response (PR) = 0

Multivariate logistic regression analysis further confirmed that PD-L1 positive expression (OR = 0.361, 95% CI: 0.161 ~ 0.808, *P* = 0.013) and ALBI <-2.68 (OR = 2.524, 95% CI: 1.148 ~ 5.552, *P* = 0.021) were independent predictors of tumor response. In the subgroup analysis of immunotherapy drugs, compared to the “other” category, camrelizumab (OR = 0.157, 95% CI: 0.042 ~ 0.590, *P* = 0.006), pembrolizumab (OR = 0.311, 95% CI: 0.109 ~ 0.885, *P* = 0.029), and sintilimab (OR = 0.258, 95% CI: 0.070 ~ 0.952, *P* = 0.042) showed statistically significant differences in efficacy. Notably, bone metastasis status and PLR, which were significant in univariate analysis, may have been confounded by other variables in multivariate analysis and did not retain statistical significance (*P* = 0.146 and *P* = 0.206, respectively).

#### Analysis of short-term efficacy factors in adenocarcinoma patients

This study analyzed 109 adenocarcinoma patients (Table 3) and found that the line of therapy and type of immunotherapy drug were significantly associated with short-term efficacy (*P* < 0.05). Patients receiving second-line therapy had the highest response rate (30.77%), while no patients on third-line therapy achieved response. The pembrolizumab group had a response rate of 50.00%, which was significantly superior to the sintilimab group (12.00%). Multivariate analysis indicated a trend toward poorer efficacy with sintilimab (OR = 5.518, *P* = 0.064). Additionally, PD-L1-positive patients had higher response rates compared to the negative group (32.14% vs. 18.52%), and patients without bone metastasis had higher response rates (27.69% vs. 13.64%), but these differences did not reach statistical significance (*P* > 0.05).

**Table 3:** Correlation Analysis Between Clinical Characteristics and Short-Term Efficacy in Adenocarcinoma Patients

Characteristic		Total	Tumor Response Group *n*(%)*	Tumor Non-Response Group *n*(%)*	x2	*P* Value	OR (95%CI)	*P* Value
Sex	Male	75	18(24.00)	57(76.00)	0.550	0.458		
	Female	34	6(17.65)	28(82.35)				
Age	<65	81	15(18.52)	66(81.48)	2.249	0.134		
	≥ 65	28	9(32.14)	19(67.86)				
ECOG Score	0 ~ 1	91	20(21.98)	71(78.02)	0.001	0.982		
	2	18	4(22.22)	14(77.78)				
Clinical Stage	Ⅲ	20	6(30.00)	14(70.00)	0.909	0.340		
	Ⅳ	89	18(20.22)	71(79.78)				
PD-L1 Expression	Negative	81	15(18.52)	66(81.48)	2.249	0.134		
	Positive	28	9(32.14)	19(67.86)				
Gene Mutation	Absent	81	15(18.18)	66(81.82)	2.249	0.134		
	Present	28	9(25.93)	19(74.07)				
Line of Therapy	1	36	8(22.22)	28(77.78)	6.266	0.044		
	2	39	12(30.77)	27(69.23)			0.971(0.309 ~ 3.052)	0.96
	3	16	0(0.00)	16(100.00)			-	0.998
	≥ 4	18	4(22.22)	14(77.78)			1.061(0.252 ~ 4.464)	0.936
Immunotherapy Drug	Other	9	4(44.44)	5(55.56)	9.862	0.043		
	Camrelizumab	40	6(15.00)	34(85.00)			3.439(0.7 ~ 16.895)	0.128
	Pembrolizumab	10	5(50.00)	5(50.00)			0.802(0.127 ~ 5.069)	0.814
	Tislelizumab	25	6(24.00)	19(76.00)			1.608(0.308 ~ 8.383)	0.573
	Sintilimab	25	3(12.00)	22(88.00)			5.518(0.908 ~ 33.515)	0.064
Treatment Regimen	Immunotherapy	24	8(33.33)	16(66.67)	3.185	0.364		
	Immuno + Chemo	62	12(19.35)	50(80.65)				
	Immuno + Targeted	4	0(0.00)	4(100.00)				
	Immuno + Chemo + Targeted	19	4(21.05)	15(78.95)				
Radiotherapy	Absent	44	7(15.91)	37(84.09)	1.604	0.205		
	Present	65	17(26.15)	48(73.85)				
Brain Metastasis	Absent	74	18(24.32)	56(75.68)	0.714	0.398		
	Present	35	6(17.14)	29(82.86)				
Bone Metastasis	Absent	65	18(27.69)	47(72.31)	3.019	0.082		
	Present	44	6(13.64)	38(86.36)				
NLR	<3.72	55	9(16.36)	46(83.64)	2.067	0.150		
	≥ 3.72	54	15(27.78)	39(72.22)				
PLR	<207.43	62	13(20.97)	49(79.03)	0.092	0.761		
	≥ 207.43	47	11(23.40)	36(76.60)				
ALBI	<-2.68	67	16(23.88)	51(76.12)	0.351	0.553		
	≥-2.68	42	8(19.05)	34(80.95)				
LDH	<226.50	43	12(27.91)	31(72.09)	1.434	0.231		
	≥ 226.50	66	12(18.18)	54(81.82)				

*Non-response (SD/PD) = 1, Response (PR) = 0

#### Analysis of short-term efficacy factors in squamous cell carcinoma patients

This study analyzed 83 squamous cell carcinoma patients (Table 4) and found that ECOG PS score (χ²=5.784, *P* = 0.016), PD-L1 expression (χ²=4.122, *P* = 0.042), NLR (χ²=8.690, *P* = 0.003), PLR (χ²=10.345, *P* = 0.001), and ALBI score (χ²=6.350, *P* = 0.012) were significantly associated with short-term efficacy. Specifically, patients with PS scores of 0–1 had significantly higher response rates than those with PS score of 2 (36.11% vs. 0%), and PD-L1-positive patients had higher response rates than the negative group (53.33% vs. 26.47%). Hematological indicator analysis showed that patients with NLR < 3.72 (45.45% vs. 15.38%), PLR < 207.43 (48.72% vs. 15.91%), and ALBI<-2.68 (42.55% vs. 16.67%) had significantly better response rates. Multivariate analysis indicated that radiotherapy showed a trend toward improved short-term efficacy (OR = 0.298, 95% CI: 0.094 ~ 0.95, *P* = 0.041), but this finding was not robust after adjustment for confounding and requires prospective validation.

**Table 4:** Correlation Analysis Between Clinical Characteristics and Short-Term Efficacy in Squamous Cell Carcinoma Patients

Characteristic		Total	Tumor Response Group *n*(%)*		Tumor Non-Response Group *n*(%)*	x2	*P* Value	OR (95%CI)	*P* Value
Sex	Male	74	23(31.08)	51(68.92)		0.019	0.891		
	Female	9	3(33.33)	6(66.67)					
Age	<65	45	17(37.78)	28(62.22)		1.902	0.168		
	≥ 65	38	9(23.68)	29(76.32)					
PS Score	0 ~ 1	72	26(36.11)	46(63.89)		5.784	0.016		
	2	11	0(0.00)	11(100.00)					
Clinical Stage	Ⅲ	37	13(35.14)	24(64.86)		0.45	0.502		
	Ⅳ	46	13(28.26)	33(71.74)					
PD-L1 Expression	Negative	68	18(26.47)	50(73.53)		4.122	0.042	0.366(0.093 ~ 1.44)	0.150
	Positive	15	8(53.33)	7(46.67)					
Gene Mutation	Absent	75	22(29.33)	53(70.67)		1.435	0.231		
	Present	8	4(50.00)	4(50.00)					
Line of Therapy	1	40	10(25.00)	30(75.00)		2.792	0.425		
	2	31	12(38.71)	19(61.29)					
	3	10	4(40.00)	6(60.00)					
	≥ 4	2	0(0.00)	2(100.00)					
Immunotherapy Drug	Other	12	4(33.33)	8(66.67)		3.062	0.548		
	Camrelizumab	9	1(11.11)	8(88.89)					
	Pembrolizumab	8	4(50.00)	4(50.00)					
	Tislelizumab	34	11(32.35)	23(67.65)					
	Sintilimab	20	6(30.00)	14(70.00)					
Treatment Regimen	Immunotherapy	9	1(11.11)	8(88.89)		7.083	0.069		
	Immuno + Chemo	70	23(32.86)	47(67.14)				0.27(0.025 ~ 2.861)	0.277
	Immuno + Targeted	2	0(0.00)	2(100.00)					
	Immuno + Chemo + Targeted	2	2(100.00)	0(0.00)					
Radiotherapy	Absent	43	10(23.26)	33(76.74)		2.701	0.1	0.298(0.094 ~ 0.95)	0.041
	Present	40	16(40.00)	24(60.00)					
Brain Metastasis	Absent	72	22(30.56)	50(69.44)		0.15	0.699		
	Present	11	4(36.36)	7(63.64)					
Bone Metastasis	Absent	66	22(33.33)	44(66.67)		0.604	0.437		
	Present	17	4(23.53)	13(76.47)					
NLR	<3.72	44	20(45.45)	24(54.55)		8.69	0.003	1.859(0.367 ~ 9.423)	0.454
	≥ 3.72	39	6(15.38)	33(84.62)					
PLR	<207.43	39	19(48.72)	20(51.28)		10.345	0.001	2.667(0.559 ~ 12.717)	0.218
	≥ 207.43	44	7(15.91)	37(84.09)					
ALBI	<-2.68	47	20(42.55)	27(57.45)		6.350	0.012	2.723(0.84 ~ 8.83)	0.095
	≥-2.68	36	6(16.67)	30(83.33)					
LDH	<226.50	36	12(33.33)	24(66.67)		0.119	0.730		
	≥ 226.50	47	14(29.79)	33(70.21)					

*Non-response (SD/PD) = 1, Response (PR) = 0

### Relationship between baseline peripheral blood biomarkers and PFS

**Fig. 3:**
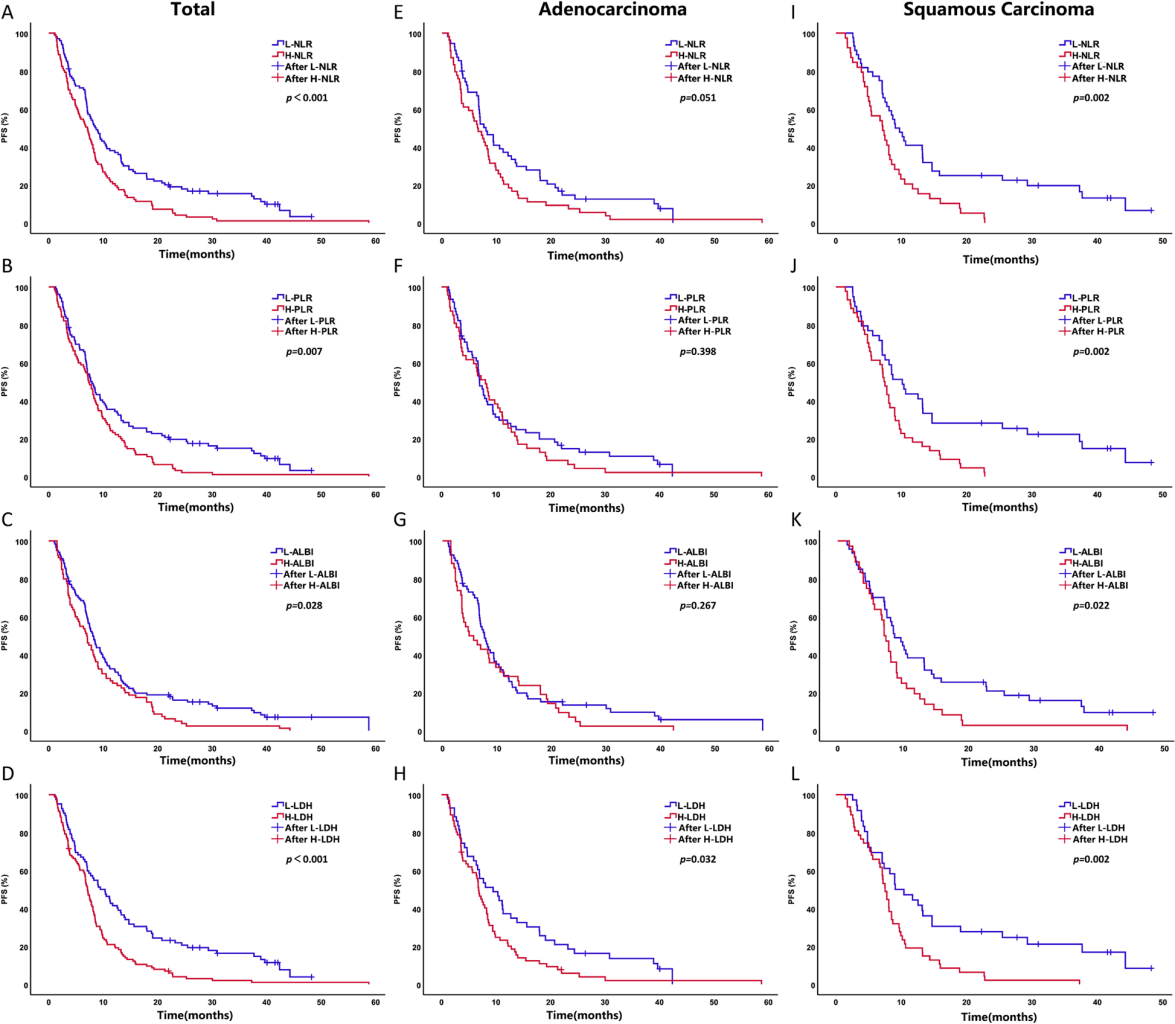
PFS Survival Curves for Patients with Different Baseline Levels of NLR, PLR, ALBI, and LDH. Kaplan–Meier curves comparing PFS between patients with low vs. high baseline levels of NLR (**A**, **E**, **I**), PLR (**B**, **F**, **J**), ALBI score (**C**, **G**, **K**), and LDH (**D**, **H**, **L**). Analyses were performed for the full cohort (**A**–**D**), the adenocarcinoma subgroup (**E**–**H**), and the squamous cell carcinoma subgroup (**I**–**L**). Cut-off values were derived from ROC analysis: NLR 3.72, PLR 207.43, ALBI -2.68, and LDH 226.5 U/L. *P*-values were calculated using the log-rank test

This study systematically evaluated the predictive value of baseline peripheral blood biomarkers on progression-free survival (PFS) in the entire cohort using Kaplan-Meier survival analysis. The results demonstrated that NLR, PLR, ALBI, and LDH all exhibited significant prognostic stratification capabilities (all *P* < 0.05). Specifically, the median PFS in the low NLR group (L-NLR) was 8.7 months (95% CI: 7.1 ~ 9.1), significantly superior to 7.1 months in the high NLR group (H-NLR) (*P* = 0.001) (Fig. 3A); the median PFS in the low PLR group was 7.9 months (95% CI: 6.6 ~ 9.2), extending by 0.5 months compared to the high PLR group (*P* = 0.007) (Fig. 3B). The median PFS in the low ALBI group was 8.1 months (95% CI: 7.1 ~ 9.1), extending by 1.1 months compared to the high ALBI group (*P* = 0.028) (Fig. 3C). Notably, LDH demonstrated the strongest predictive efficacy, with the median PFS in the low LDH group reaching 9.5 months (95% CI: 6.7 ~ 12.3), reducing the risk by 46.3% compared to the high LDH group (7.1 months, 95% CI: 6.5 ~ 7.8) (*P* < 0.001) (Fig. 3D).

In adenocarcinoma patients, the predictive patterns of peripheral blood biomarkers exhibited significant heterogeneity. LDH maintained independent prognostic value, with the median PFS in the low LDH group being 9.5 months (95% CI: 6.7 ~ 12.3), extending by 2.7 months compared to the high LDH group (*P* = 0.032) **(**Fig. 3H). However, the predictive efficacy of systemic inflammatory biomarkers was generally diminished: although the low NLR group showed a trend toward clinical benefit (8.1 vs. 6.5 months, *P* = 0.051), it did not reach statistical significance (Fig. 3E); PLR and ALBI completely lost stratification capability (both *P* > 0.05) (Figs. 3F-G).

The predictive patterns of peripheral blood biomarkers in squamous cell carcinoma patients were highly consistent with the entire cohort and demonstrated stronger prognostic associations. The median PFS in the low NLR group reached 9.1 months (95% CI: 7.0 ~ 11.2), extending by 1.9 months compared to the high NLR group (*P* = 0.002) (Fig. 3I); the prognostic advantage in the low PLR group was more pronounced (10.2 vs. 7.4 months, *P* = 0.002) (Fig. 3J). Additionally, ALBI exhibited unique predictive value in squamous cell carcinoma, with the median PFS in the low score group being 8.7 months (95% CI: 6.5 ~ 10.9), significantly superior to the high score group (7.1 months, *P* = 0.022) (Fig. 3K). LDH remained the strongest predictor, with the median PFS in the low LDH group extending by 1.6 months compared to the high LDH group (9.1 vs. 7.5 months, *P* = 0.002) (Fig. 3L).

### Relationship between baseline peripheral blood biomarkers and OS

**Fig. 4:**
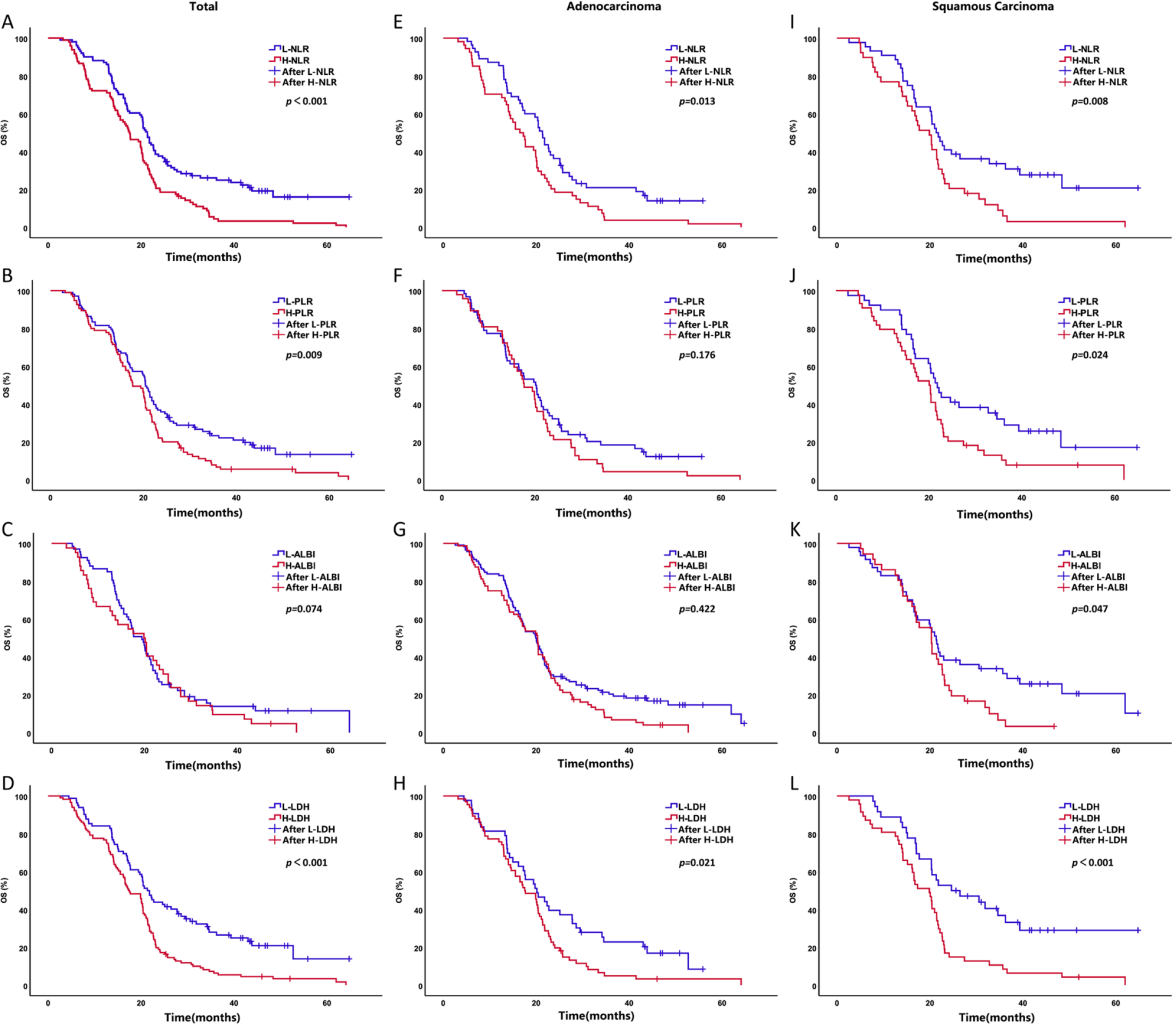
OS Survival Curves for Patients with Different Baseline Levels of NLR, PLR, ALBI, and LDH. Kaplan–Meier curves comparing OS between patients with low vs. high baseline levels of NLR (**A**, **E**, **I**), PLR (**B**, **F**, **J**), ALBI score (**C**, **G**, **K**), and LDH (**D**, **H**, **L**). Analyses were performed for the full cohort (**A**–**D**), the adenocarcinoma subgroup (**E**–**H**), and the squamous cell carcinoma subgroup (**I**–**L**). Cut-off values were derived from ROC analysis: NLR 3.72, PLR 207.43, ALBI -2.68, and LDH 226.5 U/L. *P*-values were calculated using the log-rank test

This study systematically evaluated the predictive value of baseline peripheral blood biomarkers on overall survival (OS) in the entire cohort using Kaplan-Meier survival analysis. NLR demonstrated prognostic stratification capability, with the median OS in the low NLR group being 21.4 months (95% CI: 20.1 ~ 22.6), significantly superior to 17.5 months in the high NLR group (95% CI: 14.5 ~ 20.5) (*P* < 0.001) (Fig. 4A). PLR also exhibited significant predictive value, with the median OS in the low PLR group reaching 20.4 months (95% CI: 19.1 ~ 21.8), extending by 2.7 months compared to the high PLR group (*P* = 0.009) (Fig. 4B). LDH showed excellent prognostic discrimination, with the median OS in the low LDH group being 21.4 months (95% CI: 19.2 ~ 23.5), significantly extended compared to the high LDH group (17.3 months, 95% CI: 14.5 ~ 20.2) (*P* < 0.001), reducing the risk by 42.5% (Fig. 4D). In contrast, the predictive efficacy of ALBI was relatively weaker (*P* = 0.074) (Fig. 4C).

In adenocarcinoma patients, the predictive patterns of peripheral blood biomarkers presented unique characteristics. NLR maintained significant predictive value, with the median OS in the low NLR group being 21.4 months (95% CI: 19.8 ~ 22.9), extending by 4.9 months compared to the high NLR group (*P* = 0.013) (Fig. 4E). LDH also demonstrated independent prognostic significance, with the median OS in the low LDH group reaching 20.1 months (95% CI: 14.8 ~ 25.4), significantly superior to the high LDH group (17.5 months, 95% CI: 14.0 ~ 21.1) (*P* = 0.021) (Fig. 4H). However, PLR (*P* = 0.176) and ALBI (*P* = 0.422) did not exhibit significant stratification capabilities (Figs. 4F-G).

The predictive efficacy of peripheral blood biomarkers in squamous cell carcinoma patients was more pronounced. The median OS in the low NLR group reached 21.4 months (95% CI: 18.9 ~ 23.8), significantly extended compared to the high NLR group (*P* = 0.008) (Fig. 4I). The predictive value of PLR was more evident in squamous cell carcinoma, with the median OS in the low PLR group being 21.8 months (95% CI: 19.0 ~ 24.6), extending by 1.7 months compared to the high PLR group (*P* = 0.024) (Fig. 4J). ALBI also demonstrated predictive capability in squamous cell carcinoma that was not observed in adenocarcinoma, with the median OS in the low score group being 21.4 months (95% CI: 19.2 ~ 23.6) (*P* = 0.047) (Fig. 4K). Particularly noteworthy is that LDH exhibited the strongest prognostic discrimination in squamous cell carcinoma, with the median OS in the low LDH group reaching as high as 24.6 months (95% CI: 11.7 ~ 37.6), extending by 4.8 months compared to the high LDH group (*P* = 0.001) (Fig. 4L).

### Cox regression analysis of factors associated with PFS

#### Analysis of factors influencing PFS in the full cohort

In this study, univariate and multivariate Cox proportional hazards models were used to analyze the impact of different variables on PFS (Table 5). Univariate analysis results showed that PS score (HR = 0.595, 95% CI: 0.398 ~ 0.889, *P* = 0.011) was a significant predictor of PFS, with better PS scores (0 ~ 1) significantly associated with longer PFS. Line of therapy was a partially significant predictor of PFS, with third-line therapy significantly increasing the risk of disease progression or death (HR = 1.586, 95% CI: 1.003 ~ 2.507, *P* = 0.048), while other lines (second-line, ≥fourth-line) showed no statistical difference compared to first-line therapy. Camrelizumab significantly increased the risk of disease progression (HR = 1.891, 95% CI: 1.107 ~ 3.229, *P* = 0.020), while other drugs (pembrolizumab, tislelizumab, sintilimab) showed no statistical difference compared to “other” immunotherapy drugs. Patients receiving radiotherapy had a significantly reduced risk of disease progression (HR = 0.729, 95% CI: 0.545 ~ 0.975, *P* = 0.033). All evaluated hematological function indicators were significantly associated with patient PFS (all *P* < 0.05). Specifically: patients with NLR ≥ 3.72 had a significantly increased risk of disease progression (HR = 1.647, 95% CI: 1.228 ~ 2.208, *P* < 0.001); patients with PLR ≥ 207.43 had a 49.5% increased risk (HR = 1.495, 95% CI: 1.113 ~ 2.006, *P* = 0.007). Patients with ALBI index ≥-2.68 had significantly shorter PFS (HR = 1.385, 95% CI: 1.033 ~ 1.856, *P* = 0.029). Patients with LDH ≥ 226.50 had the highest progression risk (HR = 1.811, 95% CI: 1.336 ~ 2.456, *P* < 0.001), with an 81.1% increase in risk.

**Table 5:** Univariate and Multivariate Cox Analysis of Factors Influencing PFS in the Full Cohort

Characteristic	Univariate Analysis			Multivariate Analysis		
	*P*	HR (95%CI)		*P*	HR (95%CI)	*P* for PH assumption*
Sex (Male/Female)	0.958		1.009 (0.712 ~ 1.431)			
Age(<65/≥65)	0.378		0.878(0.658 ~ 1.172)			
Pathological Type						
Other			1.00 (Reference)			
Adenocarcinoma	0.323		0.659 (0.288 ~ 1.506)			
Squamous Carcinoma	0.196		0.577 (0.250 ~ 1.329)			
PS Score(0 ~ 1/2)	0.011		0.595(0.398 ~ 0.889)	0.323	1.246(0.806 ~ 1.926)	0.303
Clinical Stage(Ⅲ/Ⅳ)	0.776		0.955(0.697 ~ 1.309)			
PD-L1 Expression (Present/Absent)	0.904		0.979(0.689 ~ 1.390)			
Gene Mutation(Present/Absent)	0.178		0.767(0.521 ~ 1.129)			
Line of Therapy						
1			1.00 (Reference)		1.00 (Reference)	
2	0.809		0.960 (0.691 ~ 1.335)	0.579	0.906(0.637 ~ 1.286)	0.988
3	0.048		1.586 (1.003 ~ 2.507)	0.124	1.499(0.895 ~ 2.509)	0.918
≥ 4	0.296		0.762(0.458 ~ 1.268)	0.031	0.506(0.272 ~ 0.940)	0.342
Immunotherapy Drug						
Other			1.00 (Reference)		1.00 (Reference)	
Camrelizumab	0.020		1.891(1.107 ~ 3.229)	0.011	2.124(1.193 ~ 3.783)	0.272
Pembrolizumab	0.841		1.069(0.555 ~ 2.059)	0.728	1.133(0.560 ~ 2.291)	0.259
Tislelizumab	0.192		1.418(0.839 ~ 2.395)	0.342	1.304(0.754 ~ 2.254)	0.197
Sintilimab	0.079		1.620(0.945 ~ 2.778)	0.151	1.496(0.863 ~ 2.594)	0.130
Treatment Regimen						
Immunotherapy			1.00 (Reference)		1.00 (Reference)	
Immuno + Chemo	0.806		1.050(0.712 ~ 1.548)	0.248	1.277(0.844 ~ 1.931)	0.338
Immuno + Targeted	0.065		2.303(0.950 ~ 5.579)	0.063	2.551(0.949 ~ 6.855)	0.204
Immuno + Chemo + Targeted	0.926		1.027(0.587 ~ 1.797)	0.277	1.422(0.754 ~ 2.684)	0.558
Radiotherapy(Present/Absent)	0.033		0.729(0.545 ~ 0.975)	0.004	0.625(0.453 ~ 0.863)	0.840
Brain Metastasis(Present/Absent)	0.896		1.023(0.730 ~ 1.433)			
Bone Metastasis(Present/Absent)	0.196		1.222 (0.902 ~ 1.655)			
NLR(≥3.72/<3.72)	<0.001		1.647(1.228 ~ 2.208)	0.011	1.642(1.119 ~ 2.408)	0.796
PLR(≥207.43/<207.43)	0.007		1.495(1.113 ~ 2.006)	0.954	0.989(0.674 ~ 1.450)	0.370
ALBI(≥-2.68/<-2.68)	0.029		1.385(1.033 ~ 1.856)	0.014	1.490(1.085 ~ 2.045)	0.779
LDH(≥226.50/<226.50)	<0.001		1.811(1.336 ~ 2.456)	< 0.001	1.821(1.312 ~ 2.526)	0.383

*P for PH assumption: derived from Pearson correlation between Schoenfeld partial residuals and rank of survival time. *P* > 0.05 supports the proportional hazards assumption

Multivariate Cox regression analysis showed that after adjusting for other confounding factors, presence of radiotherapy, NLR, ALBI, and LDH levels were independent prognostic factors influencing PFS (all *P* < 0.05). Patients receiving radiotherapy had a significantly reduced risk of disease progression (HR = 0.625, 95% CI: 0.453 ~ 0.863, *P* = 0.004); patients with NLR ≥ 3.72 had a 64.2% increased progression risk (HR = 1.642, 95% CI: 1.119 ~ 2.408, *P* = 0.011); patients with ALBI ≥-2.68 had a 49.0% increased progression risk (HR = 1.490, 95% CI: 1.085 ~ 2.045, *P* = 0.014); patients with LDH ≥ 226.50 U/L had a 82.1% increased progression risk (HR = 1.821, 95% CI: 1.312 ~ 2.526, *P* < 0.001).

#### Analysis of factors influencing PFS in adenocarcinoma patients

This study found that disease control status, gene mutation status, and LDH levels were significantly associated with PFS (Table 6). The presence of gene mutations may confer a PFS benefit (HR = 0.593, 95% CI: 0.370–0.948, *P* = 0.029), whereas patients with LDH ≥ 226.50 U/L had a significantly elevated progression risk (HR = 1.555, 95% CI: 1.035–2.334, *P* = 0.033). Additionally, NLR ≥ 3.72 demonstrated borderline significance (HR = 1.47, 95% CI: 0.995–2.172, *P* = 0.053).

**Table 6:** Univariate and Multivariate Cox Analysis of Factors Influencing PFS in Adenocarcinoma Patients

Characteristic	Univariate Analysis		Multivariate Analysis		
	*P*	HR (95%CI)	*P*	HR (95%CI)	*P* for PH assumption*
Sex (Male/Female)	0.855	1.041(0.676 ~ 1.603)			
Age(<65/≥65)	0.873	1.032(0.7 ~ 1.521)			
PS Score(0 ~ 1/2)	0.220	0.725(0.433 ~ 1.213)			
Clinical Stage(Ⅲ/Ⅳ)	0.711	1.099(0.666 ~ 1.814)			
PD-L1 Expression (Present/Absent)	0.333	0.795(0.5 ~ 1.265)			
Gene Mutation(Present/Absent)	0.029	0.593(0.37 ~ 0.948)	0.100	0.662(0.405 ~ 1.083)	0.703
Line of Therapy					
1		1.00 (Reference)			
2	0.597	0.881(0.551 ~ 1.409)			
3	0.122	1.629(0.877 ~ 3.026)			
≥ 4	0.178	0.664(0.366 ~ 1.205)			
Immunotherapy Drug					
Other		1.00 (Reference)			
Camrelizumab	0.156	1.746(0.809 ~ 3.769)			
Pembrolizumab	0.909	1.056(0.415 ~ 2.682)			
Tislelizumab	0.571	1.264(0.562 ~ 2.84)			
Sintilimab	0.177	1.745(0.778 ~ 3.913)			
Treatment Regimen					
Immunotherapy		1.00 (Reference)			
Immuno + Chemo	0.849	1.048(0.645 ~ 1.702)			
Immuno + Targeted	0.092	2.535(0.858 ~ 7.486)			
Immuno + Chemo + Targeted	0.859	1.058(0.569 ~ 1.968)			
Radiotherapy(Present/Absent)	0.133	0.738(0.497 ~ 1.097)			
Brain Metastasis(Present/Absent)	0.769	0.94(0.62 ~ 1.423)			
Bone Metastasis(Present/Absent)	0.24	1.267(0.853 ~ 1.881)			
NLR(≥3.72/<3.72)	0.053	1.47(0.995 ~ 2.172)			
PLR(≥207.43/<207.43)	0.399	1.184(0.8 ~ 1.753)			
ALBI(≥-2.68/<-2.68)	0.269	1.251(0.841 ~ 1.86)			
LDH(≥226.50/<226.50)	0.033	1.555(1.035 ~ 2.334)	0.132	1.385(0.907 ~ 2.115)	0.859

*P for PH assumption: derived from Pearson correlation between Schoenfeld partial residuals and rank of survival time. *P* > 0.05 supports the proportional hazards assumption

Notably, gene mutations (*P* = 0.100) and LDH levels (*P* = 0.132), which were significant in univariate analysis, did not maintain statistical significance in the multivariate model.

#### Analysis of factors influencing PFS in squamous cell carcinoma patients

This study indicates that performance status (PS score) and serum lactate dehydrogenase (LDH) levels are independent prognostic factors influencing progression-free survival (PFS) in patients with squamous cell carcinoma (Table 7). Univariate analysis revealed that patients with a PS score of 0–1 had a significantly lower risk of disease progression compared to those with a PS score of 2 (HR = 0.336, 95% CI: 0.169–0.67, *P* = 0.002); LDH ≥ 226.50 U/L (HR = 2.098, 95% CI: 1.293–3.404, *P* = 0.003). Additionally, hematological indicators including NLR ≥ 3.72 (HR = 2.083, *P* = 0.002), PLR ≥ 207.43 (HR = 2.115, *P* = 0.002), and ALBI score (≥-2.68, HR = 1.695, *P* = 0.024) also demonstrated statistical significance.

**Table 7:** Univariate and Multivariate Cox Analysis of Factors Influencing PFS in Squamous Cell Carcinoma Patients

Characteristic	Univariate Analysis		Multivariate Analysis		
	*P*	HR (95%CI)	*P*	HR (95%CI)	*P* for PH assumption*
Sex (Male/Female)	0.998	1.001(0.497 ~ 2.016)			
Age(<65/≥65)	0.058	0.645(0.409 ~ 1.015)			
PS Score(0 ~ 1/2)	0.002	0.336(0.169 ~ 0.670)	0.008	0.573(0.278 ~ 1.180)	0.289
Clinical Stage(Ⅲ/Ⅳ)	0.584	0.881(0.561 ~ 1.384)			
PD-L1 Expression (Present/Absent)	0.312	0.743(0.417 ~ 1.322)			
Gene Mutation(Present/Absent)	0.387	1.387(0.661 ~ 2.909)			
Line of Therapy					
1		1.00 (Reference)			
2	0.635	0.889(0.547 ~ 1.446)			
3	0.484	1.300(0.624 ~ 2.709)			
≥ 4	0.783	0.818(0.196 ~ 3.415)			
Immunotherapy Drug					
Other		1.00 (Reference)			
Camrelizumab	0.157	1.912(0.780 ~ 4.688)			
Pembrolizumab	0.852	1.095(0.424 ~ 2.828)			
Tislelizumab	0.227	1.533(0.767 ~ 3.062)			
Sintilimab	0.278	1.515(0.715 ~ 3.210)			
Treatment Regimen					
Immunotherapy		1.00 (Reference)			
Immuno + Chemo	0.917	0.961(0.455 ~ 2.031)			
Immuno + Targeted	0.477	1.770(0.367 ~ 8.524)			
Immuno + Chemo + Targeted	0.6	0.659(0.139 ~ 3.134)			
Radiotherapy(Present/Absent)	0.105	0.687(0.436 ~ 1.081)			
Brain Metastasis(Present/Absent)	0.845	1.066(0.560 ~ 2.031)			
Bone Metastasis(Present/Absent)	0.886	1.043(0.589 ~ 1.846)			
NLR(≥3.72/<3.72)	0.002	2.083(1.301 ~ 3.337)	0.602	1.193(0.614 ~ 2.321)	0.869
PLR(≥207.43/<207.43)	0.002	2.115(1.303 ~ 3.434)	0.439	1.319(0.654 ~ 2.660)	0.291
ALBI(≥-2.68/<-2.68)	0.024	1.695(1.071 ~ 2.684)	0.073	1.568(0.959 ~ 2.564)	0.397
LDH(≥226.50/<226.50)	0.003	2.098(1.293 ~ 3.404)	0.030	1.787(1.059 ~ 3.014)	0.263

*P for PH assumption: derived from Pearson correlation between Schoenfeld partial residuals and rank of survival time. *P* > 0.05 supports the proportional hazards assumption

After adjustment using a multivariate Cox proportional hazards model, LDH levels (HR = 1.787, 95% CI: 1.059 ~ 3.014, *P* = 0.030) retained independent predictive value, whereas the significance of other hematological indicators was lost.

### Cox regression analysis of factors associated with OS

#### Analysis of factors influencing OS in the full cohort

In this study, we employed univariate and multivariate Cox proportional hazards models to analyze the impact of various variables on overall survival (OS) (Table 8). Univariate analysis revealed that histological type (with other types as the reference): patients with squamous cell carcinoma exhibited a significant 62.6% reduction in mortality risk (HR = 0.374, 95% CI: 0.161–0.867, *P* = 0.022), while those with adenocarcinoma showed a borderline trend toward reduced risk (HR = 0.468, 95% CI: 0.204–1.073, *P* = 0.073), without achieving statistical significance. Patients with a PS score of 0–1 had a 47.7% lower mortality risk compared to those with a PS score of 2 (HR = 0.523, 95% CI: 0.349–0.783, *P* = 0.002). Patients with bone metastases exhibited poorer OS (HR = 1.376, 95% CI: 1.004–1.884, *P* = 0.047). Inflammation-related indicators and liver function metabolic markers were significantly associated with patient OS: patients with NLR ≥ 3.72 had a 76.8% increased mortality risk (HR = 1.768, 95% CI: 1.308–2.388, *P* < 0.001), those with PLR ≥ 207.43 had a 48.4% increased risk (HR = 1.484, 95% CI: 1.100-2.000, *P* = 0.010); meanwhile, patients with LDH ≥ 226.50 U/L showed a significant 89.9% increase in mortality risk (HR = 1.899, 95% CI: 1.388–2.599, *P* < 0.001), whereas those with ALBI ≥-2.68 displayed a trend toward increased risk (HR = 1.314, 95% CI: 0.972–1.775) that did not reach statistical significance (*P* = 0.076).

**Table 8:** Univariate and Multivariate Cox Analysis of Factors Influencing OS in the Full Cohort

Characteristic	Univariate Analysis		Multivariate Analysis		
	*P*	HR (95%CI)	*P*	HR (95%CI)	*P* for PH assumption*
Sex (Male/Female)	0.277	1.227(0.849 ~ 1.772)			
Age(<65/≥65)	0.095	0.777(0.578 ~ 1.045)			
Pathological Type					
Other		1.00 (Reference)		1.00 (Reference)	
Adenocarcinoma	0.073	0.468(0.204 ~ 1.073)	0.027	0.361(0.146 ~ 0.889)	0.696
Squamous Carcinoma	0.022	0.374(0.161 ~ 0.867)	0.021	0.350(0.143 ~ 0.854)	0.711
PS Score(0 ~ 1/2)	0.002	0.523(0.349 ~ 0.783)	0.007	0.788(0.172 ~ 1.730)	0.861
Clinical Stage(Ⅲ/Ⅳ)	0.945	0.989(0.716 ~ 1.365)			
PD-L1 Expression (Present/Absent)	0.718	0.935(0.650 ~ 1.346)			
Gene Mutation(Present/Absent)	0.080	0.692(0.459 ~ 1.045)	0.563	0.878(0.565 ~ 1.364)	0.386
Line of Therapy					
1		1.00 (Reference)			
2	0.437	0.873(0.620 ~ 1.229)			
3	0.741	1.081(0.679 ~ 1.721)			
≥ 4	0.495	0.833(0.494 ~ 1.406)			
Immunotherapy Drug					
Other		1.00 (Reference)		1.00 (Reference)	
Camrelizumab	0.073	1.647(0.954 ~ 2.842)	0.189	1.483(0.823 ~ 2.671)	0.077
Pembrolizumab	0.960	0.982(0.493 ~ 1.957)	0.943	0.974(0.475 ~ 1.997)	0.845
Tislelizumab	0.606	1.152(0.672 ~ 1.975)	0.884	1.042(0.601 ~ 1.807)	0.789
Sintilimab	0.138	1.518(0.874 ~ 2.636)	0.807	1.075(0.600 ~ 1.929)	0.280
Treatment Regimen					
Immunotherapy		1.00 (Reference)			
Immuno + Chemo	0.450	0.858(0.578 ~ 1.275)			
Immuno + Targeted	0.129	1.983(0.819 ~ 4.800)			
Immuno + Chemo + Targeted	0.561	0.843(0.473 ~ 1.501)			
Radiotherapy(Present/Absent)	0.143	0.800(0.593 ~ 1.078)			
Brain Metastasis(Present/Absent)	0.583	1.102(0.779 ~ 1.561)			
Bone Metastasis(Present/Absent)	0.047	1.376(1.004 ~ 1.884)	0.509	1.122(0.797 ~ 1.580)	0.603
NLR(≥3.72/<3.72)	<0.001	1.768(1.308 ~ 2.388)	0.032	1.536(1.037 ~ 2.275)	0.575
PLR(≥207.43/<207.43)	0.010	1.484(1.100 ~ 2.000)	0.883	0.971(0.659 ~ 1.432)	0.300
ALBI(≥-2.68/<-2.68)	0.076	1.314(0.972 ~ 1.775)	0.275	1.193(0.869 ~ 1.637)	0.246
LDH(≥226.50/<226.50)	<0.001	1.899(1.388 ~ 2.599)	0.002	1.728(1.231 ~ 2.426)	0.260

*P for PH assumption: derived from Pearson correlation between Schoenfeld partial residuals and rank of survival time. *P* > 0.05 supports the proportional hazards assumption

Multivariate Cox regression analysis indicated that, after adjusting for other confounding factors, lung cancer histological type, NLR, and LDH levels remained independent prognostic factors for OS (all *P* < 0.05). Compared to other types, patients with adenocarcinoma (HR = 0.361, 95% CI: 0.146 ~ 0.889, *P* = 0.027) and squamous cell carcinoma (HR = 0.021, 95% CI: 0.143 ~ 0.854, *P* = 0.021) had significantly reduced mortality risks by 63.9% and 65.0%, respectively; NLR ≥ 3.72 (HR = 1.536, 95% CI: 1.037 ~ 2.275, *P* = 0.032) and LDH ≥ 226.50 U/L (HR = 1.728, 95% CI: 1.231 ~ 2.426, *P* = 0.002) were associated with 53.6% and 72.8% increases in mortality risk, respectively. However, PLR (*P* = 0.856) and ALBI (*P* = 0.594) did not demonstrate statistical significance in the multivariate analysis.

#### Analysis of factors influencing OS in adenocarcinoma patients

In the adenocarcinoma patient cohort, univariate Cox regression analysis revealed that multiple clinical features were significantly associated with overall survival (OS) (Table 9). Regarding hematological indicators, both NLR ≥ 3.72 (HR = 1.651, 95% CI: 1.107–2.461, *P* = 0.014) and LDH ≥ 226.50 U/L (HR = 1.632, 95% CI: 1.073–2.482, *P* = 0.022) exhibited statistical significance. Additionally, bone metastasis status displayed borderline significance (HR = 1.447, 95% CI: 0.964–2.171, *P* = 0.075).

**Table 9:** Univariate and Multivariate Cox Analysis of Factors Influencing OS in Adenocarcinoma Patients

Characteristic	Univariate Analysis		Multivariate Analysis		
	*P*	HR (95%CI)	*P*	HR (95%CI)	*P* for PH assumption*
Sex (Male/Female)	0.236	1.306(0.840 ~ 2.030)			
Age(<65/≥65)	0.814	0.954 (0.642 ~ 1.416)			
PS Score(0 ~ 1/2)	0.115	0.661(0.395 ~ 1.107)			
Clinical Stage(Ⅲ/Ⅳ)	0.555	1.163(0.704 ~ 1.922)			
PD-L1 Expression (Present/Absent)	0.138	0.696(0.431 ~ 1.124)			
Gene Mutation(Present/Absent)	0.101	0.671(0.417 ~ 1.081)			
Line of Therapy					
1		1.00 (Reference)			
2	0.727	0.920(0.575 ~ 1.472)			
3	0.699	0.884(0.473 ~ 1.652)			
≥ 4	0.262	0.705(0.382 ~ 1.299)			
Immunotherapy Drug					
Other		1.00 (Reference)			
Camrelizumab	0.398	1.395(0.644 ~ 3.021)			
Pembrolizumab	0.960	1.025(0.394 ~ 2.667)			
Tislelizumab	0.911	1.048(0.464 ~ 2.365)			
Sintilimab	0.173	1.762(0.780 ~ 3.979)			
Treatment Regimen					
Immunotherapy		1.00 (Reference)			
Immuno + Chemo	0.435	0.823(0.505 ~ 1.341)			
Immuno + Targeted	0.463	1.495(0.511 ~ 4.378)			
Immuno + Chemo + Targeted	0.630	0.856 (0.454 ~ 1.613)			
Radiotherapy(Present/Absent)	0.358	0.828(0.554 ~ 1.238)			
Brain Metastasis(Present/Absent)	0.670	1.096 (0.718 ~ 1.673)			
Bone Metastasis(Present/Absent)	0.075	1.447(0.964 ~ 2.171)	0.230	1.292(0.850 ~ 1.963)	0.268
NLR(≥3.72/<3.72)	0.014	1.651(1.107 ~ 2.461)	0.076	1.455(0.961 ~ 2.202)	0.972
PLR(≥207.43/<207.43)	0.178	1.315(0.883 ~ 1.958)			
ALBI(≥-2.68/<-2.68)	0.423	1.178(0.789 ~ 1.760)			
LDH(≥226.50/<226.50)	0.022	1.632(1.073 ~ 2.482)	0.067	1.487(0.972 ~ 2.276)	0.186

*P for PH assumption: derived from Pearson correlation between Schoenfeld partial residuals and rank of survival time. *P* > 0.05 supports the proportional hazards assumption

Following multivariate adjustment, NLR ≥ 3.72 (HR = 1.455, 95% CI: 0.961 ~ 2.202, *P* = 0.076) and LDH levels (HR = 1.487, 95% CI: 0.972 ~ 2.276, *P* = 0.067) were reduced to borderline significance. Notably, bone metastasis, which was significant in the univariate analysis, did not maintain significance in the multivariate model (*P* > 0.1).

#### Analysis of factors influencing OS in squamous cell carcinoma patients

Univariate analysis in squamous cell carcinoma patients revealed distinct prognostic features (Table 10). Age < 65 years (HR = 0.570, 95% CI: 0.355 ~ 0.917, *P* = 0.020) and PS score of 0–1 (HR = 0.319, 95% CI: 0.163 ~ 0.626, *P* < 0.001) were associated with better OS. Hematological indicators all showed significant associations: patients with NLR ≥ 3.72 had an 89.9% increased mortality risk (HR = 1.899, 95% CI: 1.176 ~ 3.068, *P* = 0.009), PLR ≥ 207.43 had a 72.9% increased risk (HR = 1.729, 95% CI: 1.068 ~ 2.796, *P* = 0.026), ALBI ≥-2.68 had a 62.9% increased risk (HR = 1.629, 95% CI: 1.002 ~ 2.648, *P* = 0.049), and LDH ≥ 226.50 U/L had a significantly increased risk of 230.1% (HR = 2.301, 95% CI: 1.393 ~ 3.801, *P* = 0.001).

**Table 10:** Univariate and Multivariate Cox Analysis of Factors Influencing OS in Squamous Cell Carcinoma Patients

Characteristic	Univariate Analysis		Multivariate Analysis		
	*P*	HR (95%CI)	*P*	HR (95%CI)	*P* for PH assumption*
Sex (Male/Female)	0.543	1.276(0.581 ~ 2.803)			
Age(<65/≥65)	0.020	0.570(0.355 ~ 0.917)	0.843	1.059(0.603 ~ 1.858)	0.534
PS Score(0 ~ 1/2)	<0.001	0.319(0.163 ~ 0.626)	0.439	0.738(0.040 ~ 1.685)	0.787
Clinical Stage(Ⅲ/Ⅳ)	0.884	0.965(0.602 ~ 1.547)			
PD-L1 Expression (Present/Absent)	0.288	1.379(0.762 ~ 2.496)			
Gene Mutation(Present/Absent)	0.268	0.597(0.240 ~ 1.488)			
Line of Therapy					
1		1.00 (Reference)			
2	0.258	0.737(0.434 ~ 1.251)			
3	0.600	1.219(0.582 ~ 2.550)			
≥ 4	0.909	1.087(0.260 ~ 4.551)			
Immunotherapy Drug					
Other		1.00 (Reference)			
Camrelizumab	0.115	2.118(0.833 ~ 5.388)			
Pembrolizumab	0.924	0.952(0.342 ~ 2.647)			
Tislelizumab	0.569	1.234(0.599 ~ 2.545)			
Sintilimab	0.535	1.279(0.588 ~ 2.784)			
Treatment Regimen					
Immunotherapy		1.00 (Reference)		1.00 (Reference)	
Immuno + Chemo	0.962	1.019(0.465 ~ 2.235)	0.473	1.354(0.592 ~ 3.096)	0.971
Immuno + Targeted	0.062	4.652(0.925 ~ 23.399)	0.007	12.005(1.951 ~ 73.857)	0.645
Immuno + Chemo + Targeted	0.349	0.349(0.045 ~ 2.987)	0.753	0.706(0.081 ~ 6.143)	0.561
Radiotherapy(Present/Absent)	0.116	0.682(0.423 ~ 1.099)			
Brain Metastasis(Present/Absent)	0.915	1.039(0.514 ~ 2.100)			
Bone Metastasis(Present/Absent)	0.871	1.050(0.584 ~ 1.889)			
NLR(≥3.72/<3.72)	0.009	1.899(1.176 ~ 3.068)	0.334	1.409(0.703 ~ 2.825)	0.392
PLR(≥207.43/<207.43)	0.026	1.729(1.068 ~ 2.796)	0.660	0.851(0.414 ~ 1.747)	0.775
ALBI(≥-2.68/<-2.68)	0.049	1.629(1.002 ~ 2.648)	0.059	1.664(0.981 ~ 2.824)	0.060
LDH(≥226.50/<226.50)	0.001	2.301(1.393 ~ 3.801)	0.004	2.372(1.327 ~ 4.238)	0.909

*P for PH assumption: derived from Pearson correlation between Schoenfeld partial residuals and rank of survival time. *P* > 0.05 supports the proportional hazards assumption

The multivariate model showed that LDH ≥ 226.50 U/L was an independent prognostic factor for OS (HR = 2.372, 95% CI: 1.327 ~ 4.238, *P* = 0.004). However, age, PS score, and inflammatory indicators that were significant in univariate analysis did not retain independence (all *P* > 0.05).

### Analysis of immune-related adverse events

This study conducted a systematic analysis of irAEs in 198 patients with advanced NSCLC receiving immunotherapy (Table 11). The results showed that 92 patients (46.46%) experienced irAEs of varying degrees, Non-hematological toxicities were primarily gastrointestinal reactions (nausea/vomiting 32.32%, abdominal pain/diarrhea 25.25%) and dermatological toxicities (rash 23.74%, pruritus 25.25%), but severe events (grade III and above) were rare (all ≤ 2.53%). Notably, the overall incidence of immune-related pneumonia was 7.07%, with 42.9% (6/14) being grade III and above events. In terms of endocrine toxicities, the incidence of thyroid dysfunction was 21.21%, with 16.7% (7/42) being grade III and above events.

**Table 11:** irAEs in Advanced NSCLC Patients Receiving Immunotherapy

Adverse Event	Grade I-II (%)	Grade III and Above (%)	Total Incidence (%)
Total	69(34.85)	23(11.6)	92(46.46)
Nausea/Vomiting	64 (32.32)	0 (0.00)	64 (32.32)
Abdominal Pain/Diarrhea	50 (25.25)	0 (0.00)	50 (25.25)
Rash	47 (23.74)	0 (0.00)	47 (23.74)
Pruritus	45 (22.73)	5 (2.53)	50 (25.25)
Liver Function Abnormality	35 (17.68)	9 (4.55)	44 (22.22)
Immune-Related Pneumonia	8 (4.04)	6 (3.03)	14 (7.07)
Immune-Related Myocarditis	0 (0.00)	0 (0.00)	0 (0.00)
Thyroid Dysfunction	35 (17.68)	7 (3.53)	42 (21.21)

Further analysis of the association between hematological indicators and irAEs revealed that NLR and LDH were significantly correlated with irAEs risk (Table 12). The irAEs incidence in the low NLR group (< 3.72) was significantly higher than in the high NLR group (55.4% vs. 37.1%, *P* = 0.010), and patients with low NLR had nearly twice the risk of developing irAEs (OR = 1.976, 95% CI: 1.098 ~ 3.557, *P* = 0.023). Similarly, the irAEs incidence in the low LDH group (< 226.50 U/L) was significantly higher than in the high LDH group (62.2% vs. 35.3%, *P* < 0.001), with the risk increased nearly threefold (OR = 2.881, 95% CI: 1.590 ~ 5.223, *P* < 0.001). In contrast, PLR and ALBI showed no significant statistical association with irAEs (*P* = 0.143 and *P* = 0.528).

**Table 12:** Correlation Analysis Between Hematological Indicators and irAEs

Hematological Indicator		Total	irAEs Group *n*(%)	Non-irAEs Group *n*(%)	x^2^	*P* Value	OR (95%CI)	*P* Value
NLR	<3.72	101	56(55.4)	45(44.6)	6.685	0.010	1.976(1.098 ~ 3.557)	0.023
	≥ 3.72	97	36(37.1)	61(62.9)				
PLR	<207.43	103	53(51.5)	50(48.5)	2.150	0.143		
	≥ 207.43	95	39(41.1)	56(58.9)				
ALBI	<-2.68	118	57(48.3)	61(51.7)	0.398	0.528		
	≥-2.68	80	35(43.8)	45(56.3)				
LDH	<226.50	82	51(62.2)	31(37.8)	13.923	<0.001	2.881(1.590 ~ 5.223)	<0.001
	≥ 226.50	116	41(35.3)	75(64.7)				

## Discussion

Although the precision of NSCLC treatment has significantly improved in recent years, it remains challenging. The emergence of PD-1 inhibitors has provided new hope for advanced NSCLC patients, but many clinical studies indicate that only a minority benefit [31]. Therefore, identifying effective predictive biomarkers to screen potential beneficiaries is urgent. This study explored the relationship between baseline peripheral blood biomarkers (NLR, PLR, ALBI, LDH) and short-term efficacy, PFS, and OS in advanced NSCLC patients, further analyzing their prognostic value. Our results show that NLR, PLR, ALBI, and LDH have significant roles in predicting PFS and OS, and combined analysis may enhance their clinical utility. Additionally, this study revealed significant pathological subtype (squamous vs. adenocarcinoma) influences on biomarker predictive efficacy, providing new perspectives for individualized stratification.

This study, by analyzing baseline blood biomarkers in advanced NSCLC patients, found that NLR, PLR, ALBI, and LDH have significant meaning in predicting efficacy and survival. ROC curve analysis showed AUC values for NLR, PLR, ALBI, and LDH of 0.804, 0.694, 0.684, and 0.726 (Fig. 2), with NLR and LDH having the best predictive efficacy (AUC > 0.70), suggesting that systemic inflammation (NLR/PLR) and tumor metabolic activity (LDH) may jointly drive immunotherapy response differences.

This study further clarified key factors influencing immunotherapy short-term efficacy (ORR). Multivariate logistic regression showed PD-L1 positive expression (OR = 0.361, *P* = 0.013) and ALBI<-2.68 (OR = 2.524, *P* = 0.021) as independent predictors of objective response, suggesting synergistic roles of tumor immunogenicity and liver function metabolic status in determining early treatment response. Notably, although PD-L1-positive patients had significantly higher ORR (40.91% vs. 21.43%), their protective effect in multivariate models (OR < 1) may reflect the dynamic complexity of PD-L1 expression—some positive patients have weakened benefits due to high tumor burden or immunosuppressive microenvironments. The predictive value of PLR < 207.43 (OR = 1.892, *P* = 0.035) and low ALBI score (OR = 2.524, *P* = 0.021) further supports the key roles of systemic inflammation and liver function in early immune activation.

Kaplan-Meier survival analysis further revealed that lower NLR (< 3.72), PLR (< 207.43), ALBI (<-2.68), and LDH (< 226.5 U/L) levels were associated with longer PFS and OS. Among them, LDH’s stratification ability was most prominent, with low-level group median PFS extended by 2.4 months (9.5 vs. 7.1 months), and OS risk reduced by 82.6% (HR = 1.826), consistent with LDH-mediated lactate metabolism inhibiting T cell function and promoting immune escape. Further subgroup analysis showed that biomarker predictive value has significant pathological type dependency: in squamous carcinoma patients, low NLR (< 3.72) and low PLR (< 207.43) extended median PFS by 1.9 months (*P* = 0.002) and 2.8 months (*P* = 0.002), respectively, while in adenocarcinoma, only LDH retained independent prognostic value (*P* = 0.032). This difference may stem from stronger inflammation-driven features in squamous carcinoma microenvironments (e.g., high IL-6/TNF-α expression [32]), while adenocarcinoma’s metabolic heterogeneity (e.g., EGFR/KRAS mutation-related glycolysis reprogramming [33]) may weaken inflammatory marker predictive efficacy. Additionally, ALBI score’s independent prognostic role in PFS and OS was validated, with lower ALBI scores (<-2.68) associated with longer survival, suggesting liver function not only affects drug metabolism and clearance but may also indirectly enhance immune response by regulating systemic inflammation balance (e.g., albumin maintaining vascular permeability, bilirubin antioxidative stress).

Inflammatory status is closely related to the prognosis of various cancers [32, 33], and NLR and PLR evaluated in this study are good biomarkers reflecting inflammatory status. In a clinical study by Bagley et al. [10], analysis of 175 advanced NSCLC patients treated with nivolumab showed baseline NLR ≥ 5 as a poor prognostic factor for OS (HR = 1.83, 95% CI:1.2 ~ 2.8, *P* = 0.006) and an independent predictor for PFS (HR = 1.42, 95% CI:1.02 ~ 2.0, *P* = 0.04). Another retrospective study also indicated that baseline NLR > 5 was closely related to poor OS [31]. A meta-analysis by Cao [25] et al. included 14 retrospective studies involving 1225 NSCLC patients, exploring the predictive value of baseline and post-treatment NLR for nivolumab efficacy. Pooled results showed higher baseline NLR associated with poorer PFS (HR = 1.44, 95% CI:1.18 ~ 1.77, *P* < 0.05) and OS (HR = 1.75, 95% CI:1.33 ~ 2.30, *P* < 0.05). Subgroup analysis further indicated NLR ≥ 5 was more reliable for predicting PFS (HR = 1.73, 95% CI:1.14 ~ 2.62, *P* < 0.05) and OS (HR = 1.76, 95% CI:1.47 ~ 2.10, *P* < 0.05). Additionally, post-treatment NLR elevation was associated with poorer PFS (HR = 3.17, 95% CI:1.48 ~ 6.82, *P* < 0.05) and OS (HR = 2.26, 95% CI:1.05 ~ 4.86, *P* < 0.05). These results suggest baseline and post-treatment NLR as potential prognostic markers for NSCLC patients receiving nivolumab. Zhou [34] et al.‘s meta-analysis included 21 studies covering 2312 advanced lung cancer patients receiving immunotherapy. Pooled analysis showed elevated PLR associated with poorer OS (HR = 2.24, 95% CI:1.87 ~ 2.68, I²=44%, *P* = 0.01) and PFS (HR = 1.66, 95% CI:1.36 ~ 2.04; I²=64%, *P* < 0.01). Zhang Na [35] et al. included 1845 NSCLC patients from 21 studies, with overall analysis showing high NLR significantly associated with poorer OS (HR = 2.50, 95% CI:1.79 ~ 3.51, *P* < 0.001) and PFS (HR = 1.77, 95% CI:1.51 ~ 2.01, *P* < 0.001). Subgroup results were consistent. Similarly, pooled PLR results showed elevated PLR associated with poorer OS (HR = 1.93, 95% CI:1.51 ~ 2.01, *P* < 0.001) and PFS (HR = 1.57, 95% CI:1.30 ~ 1.90, *P* < 0.001). In our study, NLR was an independent prognostic factor for OS and PFS, with higher NLR (NLR ≥ 3.72) significantly associated with poorer OS (HR = 1.629, 95% CI:1.099 ~ 2.415, *P* = 0.015) and PFS (HR = 1.638, 95% CI:1.117 ~ 2.402, *P* = 0.012). Similarly, high baseline PLR group showed poorer OS and PFS, but not significantly in multivariate analysis.

LDH is a metabolic enzyme secreted by proliferative tumor cells, with serum levels often used as a biomarker for tumor burden. Multiple studies show LDH significantly correlates with prognosis in melanoma patients receiving immune checkpoint inhibitors [36, 37]. Reports have explored LDH in predicting PFS [38, 39] and OS [40] in ICIs-treated NSCLC patients. In Taniguchi et al. [38]’s retrospective study of 201 advanced NSCLC patients receiving nivolumab, baseline LDH > 240 U/L was significantly associated with poorer PFS (*P* < 0.05). In L. Mezquita [40]’s study, patients with LDH exceeding the upper normal limit had HR = 2.51 for OS (95% CI:1.32 ~ 4.76) compared to low LDH, indicating LDH as an important independent prognostic factor for advanced lung cancer patients receiving ICIs. This study also confirmed baseline LDH levels significantly associated with PFS and OS in ICIs-treated advanced NSCLC patients, with low LDH group PFS at 9.5 months (vs. 7.1 months, *P* < 0.001), OS risk reduced by 82.6% (HR = 1.826, *P* = 0.001). Similar results were seen in adenocarcinoma and squamous subgroups: in adenocarcinoma, low LDH group PFS extended by 2.7 months (*P* = 0.032), OS by 2.6 months (*P* = 0.021). In squamous patients, low LDH group OS reached 24.6 months (vs. 19.8 months, *P* = 0.001), with PFS also significantly extended (*P* = 0.017). Meanwhile, LDH levels were significantly associated with irAEs, with low LDH group (< 226.50 U/L) having higher irAEs incidence (62.2% vs. 35.3%, *P* < 0.001) and risk (OR = 2.881, 95% CI:1.590 ~ 5.223, *P* < 0.001).

Additionally, the application of ALBI grading in assessing immunotherapy prognosis has gradually gained attention. Pinato and Kaneko et al. have explored ALBI grading’s prognostic value in hepatocellular carcinoma patients receiving immunotherapy [41]. They found pre-treatment ALBI grading could predict OS extension and was superior to Child-Pugh grading in predicting mortality. Kinoshita et al. compared pre-operative ALBI grading with clinicopathological features and prognosis in resectable NSCLC patients, finding ALBI grades 2/3 associated with poor prognosis [30]. Ryosuke Matsukane et al. [42]’s study showed ALBI grading as a pre-treatment liver reserve assessment could significantly predict survival in ICIs-treated NSCLC patients, with pre-treatment ALBI grading as independent prognostic factor for PFS (HR 0.57, 95% CI 0.38 ~ 0.86, *p* = 0.007) and OS (HR = 0.45, 95% CI:0.29 ~ 0.72, *P* = 0.001), indicating ALBI grading-evaluated pre-treatment liver function as an important biomarker for predicting ICIs efficacy in NSCLC. This article determined the optimal ALBI cut-off as -2.68 via calculation; in the full cohort, ALBI<-2.68 group PFS extended by 1.6 months (8.7 vs. 7.1 months, *P* = 0.022), OS by 3.9 months (21.4 vs. 17.5 months, *P* = 0.047), and multivariate analysis proved ALBI as independent predictor for PFS and OS. Similarly, in squamous patients, low ALBI score group median OS reached 21.4 months (vs. 17.3 months, *P* = 0.047), with significant independent predictive value.

The optimal cutoffs for NLR (3.72), PLR (207.43), ALBI (–2.68), and LDH (226.5 U/L) were derived using time-dependent ROC analysis with the Youden index in our cohort. These values differ slightly from some literature-reported thresholds (e.g., NLR often dichotomized at 3 or 5). This variability is expected due to cohort heterogeneity, differences in disease stage, treatment regimens, and statistical methodology.

This study also evaluated clinical factors like PS score on patient prognosis via Cox regression. Results showed PS score significantly influenced PFS and OS. Poorer PS scores (PS ≥ 2) were closely associated with shorter PFS and OS, validating PS score as a classic prognostic indicator in lung cancer.

This study provides valuable insights into exploring baseline blood biomarkers’ prognostic value in immunotherapy for advanced NSCLC patients but has limitations. First, as a retrospective analysis from two centers (Renmin Hospital of Wuhan University and Macheng People’s Hospital Tumor Centers), although multi-center design partially reduced single-institution bias, results may be influenced by inter-center data collection standard differences, regional variations, patient selection bias, and data completeness limitations. Second, all subgroup analyses were exploratory; no adjustment for multiple comparisons was applied. Results should be interpreted as hypothesis-generating and require confirmation in independent cohorts. Additionally, while we analyzed multiple biomarkers (e.g., NLR, PLR, ALBI, LDH), this study did not deeply explore other factors potentially affecting immunotherapy prognosis, such as tumor gene mutations, immune microenvironment, and regimen differences, which may play varying roles in different patient groups. Finally, although we validated significant relationships between ALBI, NLR, PLR, etc., and PFS/OS, their clinical operability and practical application need further exploration and optimization, especially how to efficiently apply these markers in real clinical settings for efficacy prediction.

## Conclusion

This study found that peripheral blood markers (NLR, PLR, ALBI, LDH) have important predictive value for survival outcomes in advanced NSCLC, with significant pathological subtype heterogeneity: squamous prognosis mainly driven by systemic inflammation, while adenocarcinoma depends more on metabolic activity and molecular features, suggesting the need for subtype-specific biomarker strategies. Short-term disease control status is a key predictor of survival, radiotherapy improves prognosis, but immuno-combined targeted therapy may increase mortality risk in squamous patients. Meanwhile, baseline NLR and LDH are significantly associated with irAEs risk. These findings provide a theoretical basis for integrating inflammation, metabolism, and treatment response markers to build stratification models, hoping to guide clinical decisions through multi-dimensional biomarkers, optimizing treatment selection while balancing efficacy and safety, advancing precise practice of individualized immunotherapy in advanced NSCLC.

## Supplementary Information

Supplementary Material 1. Supplementary Table S1 Gene mutation status of the 198 enrolled patients.

## Data Availability

The data used and analysed in this study are available from the corresponding author on reasonable request.

## Abbreviations

ALB: Albumin
ALBI: Albumin-bilirubin
ALC: Absolute Lymphocyte Count
AMC: Absolute Monocyte Count
ANC: Absolute Neutrophil Count.
AST: Aspartate Aminotransferase
AUC: Area Under The Curve
CR: Complete Response
DCR: Disease Control Rate
ECOG PS: Eastern Cooperative Oncology Group Performance Score
ICIs: Immune Checkpoint Inhibitors
irAEs: Immune-related Adverse Events
LDH: Lactate dehydrogenase
NLR: Neutrophil-to-lymphocyte Ratio
NSCLC: Non-Small Cell Lung Cancer
OS: Overall Survival
ORR: Overall Response Rate
PFS: Progression Free Survival
PR: Partial Response
PD: Progressive Disease
PD-1: Programmed Death 1
PD-L1: Programmed Death-ligand 1
PLR: Platelet-to-lymphocyte Ratio
ROC: Receiver Operating Characteristic
TME: Tumor microenvironment
