# Supplementary material for: Characteristics and hematological indicators predictors of immunotherapy response in advanced non-small cell lung cancer
Source: BMC Cancer. 2026 Jun 11;26:974. doi: 10.1186/s12885-026-16302-w (PMC13471613; doi:10.1186/s12885-026-16302-w)
Supplement: Supplementary file 1 [file 12885_2026_16302_MOESM1_ESM.docx]

**Supplementary Table S1**

Gene mutation status of the 198 enrolled patients.

|  | | Tumor Response | Tumor Non-Response | Total |
| --- | --- | --- | --- | --- |
| Gene Mutation | Absent | 38 | 124 | 162 |
|  | KRAS | 3 | 2 | 5 |
|  | EGFR | 5 | 8 | 13 |
|  | MET | 0 | 3 | 3 |
|  | HER-2 | 0 | 3 | 3 |
|  | ALK | 4 | 2 | 6 |
|  | Other | 1 | 5 | 6 |
| Total | | 51 | 147 | 198 |
